# Increased Effect Sizes in a Mindfulness- and Yoga-Based Intervention After Adjusting for Response Shift with Then-Test

**DOI:** 10.1007/s12671-023-02102-x

**Published:** 2023-03-16

**Authors:** L. Javier Bartos, M. Pilar Posadas, Wendy Wrapson, Chris Krägeloh

**Affiliations:** 1 Department of Psychology and Neuroscience, Auckland University of Technology, 90 Akoranga Drive, Auckland, 1142 New Zealand; 2 Department of Pedagogy and Singing, Royal Conservatory of Music Victoria Eugenia, 46 San Jerónimo Street, 18001 Granada, Spain; 3 Faculty of Education, Camilo Jose Cela University, 11 Marqués del Riscal Street, 28010 Madrid, Spain; 4 School of Clinical Sciences, Auckland University of Technology, 90 Akoranga Drive, Auckland, 1142 New Zealand

**Keywords:** Mindfulness, Response shift, Effect size, Adjusted change, Then-test, Student musicians, CRAFT program

## Abstract

**Objectives:**

Response shift refers to variations in self-reported evaluations at different times from changes in one’s internal standards, values, and meanings. The current study explored the utility of the *then-test* to detect a potential mindfulness-based response shift occurrence during a mindfulness- and yoga-based intervention for student musicians, and to ascertain to what extent effect sizes could differ when adjusting for it.

**Method:**

Participants (*n* = 31) completed the Five Facet Mindfulness Questionnaire (FFMQ) halfway through the intervention (Time 1-FFMQ), post-intervention (Time 2-FFMQ), and immediately after Time 2-FFMQ with a then-test approach that asked participants to rate the FFMQ based on retrospective reflections on their mindfulness at Time 1 (then-test-FFMQ). Paired *t*-tests and Hedges’ *g* effect sizes were computed to estimate three potential effects: response shift (Time 1-FFMQ minus then-test-FFMQ), the conventional intervention effect (Time 2-FFMQ minus Time 1-FFMQ), and the effect after adjusting for response shift (i.e., actual intervention effect = Time 2-FFMQ minus then-test-FFMQ).

**Results:**

Response shift was significant for the FFMQ Observe subscale (*g* = 0.41) and total scale (*g* = 0.37). The adjusted scores in all subscales (Observe, *g* = 0.47; Describe, *g* = 0.25; Act Aware, *g* = 0.40; Non-judge, *g* = 0.28; Non-react, *g* = 0.57) and total scale (*g* = 0.60) achieved significance and yielded larger effect sizes than the conventional results, for which only Act Aware (*g* = 0.28), Non-react (*g* = 0.36), and total scale (*g* = 0.28) were significant.

**Conclusions:**

Notwithstanding some methodological limitations, this study lends support to the utility of the then-test to quantify response shift. When adjusting for it, effect sizes from a mindfulness- and yoga-based intervention were generally amplified.

**Preregistration:**

This study was not preregistered.

The theoretical foundations of response shift can be traced back nearly 50 years to researchers questioning the validity of pre-post self-reported instruments to measure the effects of interventions in educational training (Howard & Dailey, 1979; Howard et al., 1979) and organization development (Golembiewski et al., 1975). Howard et al. (1979) conceptualized response shift as pre-post self-reported changes in participants’ internal standards of measurement of a particular measured construct from training. Howard et al. (1979) also highlighted that such a response shift effect could be potentially intensified if the aim of the intervention is to modify participants’ conception or awareness of the construct being measured. Earlier, Golembiewski et al. (1975) had posited similar ideas relating to plausible levels of intra-subject variation masking the actual outcome and/or interpretation derived from pre-post self-reported instruments.

While the so-called Alpha change (Golembiewski et al., 1975) is understood as the theoretical intervention effect (i.e., post-test minus pre-test scores), this effect is often confounded by the presence of other types of change. Golembiewski et al. (1975) explained Beta change as an internal recalibration effect in response to a certain experience (e.g., exposure to organizational development interventions). As a result of this experience, an individual’s psychological distance between the rating scale intervals used to measure a given construct domain may have changed, thus affecting how the construct is assessed by the self-reported instrument. For instance, a participant’s self-reported score in teamwork skills of “Good” before a teambuilding intervention may remain “Good” after it, despite experiencing an actual improvement in teamwork skills. This effect may occur because the intervention might make the participant recalibrate their notion of good teambuilding skills. The initial rating of “Good” was in actuality anchored at a lesser level from the perspective of the new internal standard.

Another relevant bias described by Golembiewski et al. (1975) was Gamma change, defined as a substantial shift in an individual’s perceptual frame of reference, also induced by a certain experience, entailing the reconceptualization of certain dimensions of reality. As explained by the authors, Gamma change would involve a profound change of perspective that could be reflected in an out-of-the-scale outcome that self-reported instruments would fail to register and even confound (Golembiewski et al., 1975). For example, a participant following a holistic well-being intervention with an emphasis on spiritual development (e.g., yoga and mindfulness programs) might experience a major change in the way they conceive disease: from an initial understanding of it as a problem or negative event to a rather positive process and opportunity to grow. Similarly, their conception and approach to health, well-being, and quality of life might be drastically changed from participating in such interventions (Desikachar et al., 2005; Sullivan et al., 2018). In contrast to both Beta and Gamma change, either occurrence of which could be representative of response shift, in Alpha change (i.e., the traditionally recognized pre-post change), it is assumed that the individual’s frame of reference and psychological distance between the rating scale intervals remain constant from pre- to post-intervention (Golembiewski et al., 1975).

Over the last two decades, the response shift phenomenon has been particularly investigated in health-related quality of life and clinical intervention studies to shed light on unexpected self-reported outcomes, such as chronically ill patients reporting the same or higher quality of life than healthier individuals (Albrecht & Devlieger, 1999; Breetvelt & Dam, 1991; Rapkin & Schwartz, 2004). Schwartz and Sprangers (1999) and Sprangers and Schwartz (1999), inspired by Howard et al. (1979) and Golembiewski et al. (1975), explained the response shift phenomenon based on recalibration (i.e., change in internal standards or Beta change) and reconceptualization (i.e., Gamma change), to which they added a third component, namely reprioritization. Reprioritization, which was already implicit within the Gamma change conception of Golembiewski et al. (1975), was explained as a change in one’s values and priorities in terms of how the relevance of aspects of one’s life relative to others varies after particular events (Schwartz & Sprangers, 1999; Sprangers & Schwartz, 1999). For instance, after being diagnosed with a major disease, an individual, whose main priorities may have been on accruing professional accomplishments, may diametrically shift to nurturing core human values (e.g., altruism, love, compassion, respect, sympathy). Thus, as a result of such values becoming more salient to the individual, they may experience an equal or even greater quality-of-life experience than before the diagnosis, contrary to what normally would be expected if no information about the person’s behavior according to their reprioritization was furnished. Therefore, with the inclusion of reprioritization, Schwartz and Sprangers (1999) and Sprangers and Schwartz (1999) provided the first multifactorial definition of response shift. According to the authors, response shift refers to variations in the conception of one’s self-assessment at different times due to changes in one’s internal standards of measurement (i.e., recalibration), values (i.e., reprioritization), and meanings (i.e., reconceptualization) of a construct when confronted with particular catalysts (e.g., individual health-related changes due to disease or treatment).

In recent years, mindfulness- and yoga-based interventions have increased in popularity with numerous studies evidencing the efficacy of these two approaches for bestowing a broad spectrum of physical, emotional, cognitive, and health and well-being benefits in a large range of populations, settings, and contexts (Breedvelt et al., 2019; Corbally & Wilkinson, 2021; Ciezar-Andersen et al., 2021; Field, 2016; Garrote-Caparrós et al., 2021; Gothe et al., 2019; Halladay et al., 2019; Liu et al., 2021; Mercado et al., 2021; Sivaramakrishnan et al., 2019). Although many of the outcomes yielded from mindfulness- and yoga-based interventions, either significant or not, have been gathered through performance-based measures, the employment of self-reported instruments has frequently been the predominant method for many others (e.g., health-related quality of life, psychological distress, and dispositional mindfulness; Medvedev et al., 2022), which tend to be susceptible to response shift (Sauer et al., 2013). Particularly with reference to self-evaluation of mindfulness, for which psychometric scales have been the gold standard instruments, various authors have identified response shift as a potential bias limiting the validity of these methods (Grossman, 2008; Krägeloh et al., 2018; Sauer et al., 2013). These authors surmised that participants’ frame of reference for their mindfulness understanding and skill when responding to a mindfulness inventory might differ from pre- to post-intervention as a result of mindfulness training. According to Sprangers and Schwartz’s (1999) response shift definition, provided it were extrapolated to a mindfulness context where a mindfulness intervention functioned as a catalyst, such changes could translate into a potential recalibration and reconceptualization mindfulness-induced response shift occurrence.

The following hypothetical case illustrates how such a mindfulness-based response shift effect may occur: A female participant with no previous mindfulness experience rates the fourth item of the Five Facet Mindfulness Questionnaire (FFMQ; Baer et al., 2006) — “I perceive my feelings and emotions without having to react to them” — before and after a mindfulness-based intervention. It could be argued that, before the intervention, she might not have had a clear experiential understanding of what a feeling or an emotion is, nor the difference between the two, nor her awareness of their perception, nor the ability of non-reacting to them upon realizing them. From such a beginner’s frame of reference and internal standards, it could be contended that, due to her inexperience with the practical and conceptual implications of the item, she could overestimate her rating by answering, for instance, “sometimes true.” However, during the mindfulness training — embarking upon a somewhat reconceptualization-based learning process — she experientially develops her awareness and understanding of emotions and feelings as well as her capacity for perceiving them without reacting. Additionally, she realizes not only that her non-reactivity skills were lower than she initially thought — for she is more aware of her emotional reactions — but also that she is enhancing them over time. Therefore, the participant’s internal standards would have changed to a more experienced frame of reference from which more stringent self-evaluations might occur (i.e., higher standards). Accordingly, when asked to rate the same item again after the intervention, she might gauge it as equal or even lower than before the training. As a result, such a potential recalibration and/or even reconceptualization mindfulness-based response shift occurrence would veil the actual pre-post improvement that the participant might have both perceived and gone through. In turn, it could be contended that this concealed enhancement could be the expression of what in signal detection theory is designated as a *miss* response (Lynn & Barrett, 2014) insofar as there was a stimulus (i.e., an improvement), but it could not be detected through the participant-instrument interaction due to response shift.

Thus, if the above-mentioned particular case could be generalizable to other FFMQ items (or even other self-reported outcome measures completed before and after a mindfulness-based intervention), the following two scenarios could be postulated in the context of a *miss* response due to response shift: no effect detectable by the outcome measure, when there was actually an underlying effect, and some limited effect detected by the measure, although the underlying effect was larger. In this vein, in the extant literature of mindfulness-based interventions, there have been researchers who have found either improvements in mindfulness scores but no changes in other outcome measures (Hopkins & Proeve, 2013; Simons, 2014), improvements in other outcome measures but no effect or inconsistent results in mindfulness (Ainsworth et al., 2013; De Vibe et al., 2013; Gockel et al., 2013; Himelstein et al., 2015), or even a null effect in both (Damião Neto et al., 2019; Horner et al., 2014; Kaplan et al., 2022; Spragg, 2011). Could this contradictory, mixed, and/or unexpected findings be hinging to a certain degree on the response shift phenomenon?

Considering the aforementioned evidence and plausible outcome-related scenarios potentially affected by a response shift occurrence, it is not surprising that previous researchers in the field had already underlined the need to investigate this phenomenon for its potentiality to drive measurement error and, in doing so, look for solutions to correct or minimize it (Grossman, 2008; Krägeloh et al., 2018; Sauer et al., 2013). In this regard, though still from a field in its early infancy, previous work using measurement invariance testing (Krägeloh et al., 2018) suggests that response shift may indeed be a potential bias for confounding mindfulness measurement. However, further research relying on other methods has been recommended, particularly considering the shortcomings of former approaches, such as requiring intricate statistical analysis and large sample sizes (Finkelstein et al., 2014; Krägeloh et al., 2018). One of such recommended methods is the so-called *then-test* (Finkelstein et al., 2014; Howard et al., 1979; Sprangers et al., 1999). The then-test, or retrospective pre-test/post-test design, has been the most typically employed method to detect response shift over the years by the primary developers of the field, comprising educational (Howard & Dailey, 1979; Howard et al., 1979) and quality-of-life (Finkelstein et al., 2014; Schwartz & Sprangers, 2010; Sprangers et al., 1999; Visser et al., 2005) researchers.

A then-test questionnaire is typically administered immediately after participants complete its counterpart post-test survey, with instructions for participants to re-evaluate the same post-test items retrospectively, that is to say, thinking back at how the content of those items was applicable to them at the time of the pre-test (Schwartz & Sprangers, 2010). In such a way, the then-test would be the equivalent of completing a pre-test again but at the time of the post-test. Therefore, this procedure would allow participants to rate their tendencies or level of functioning about a particular construct domain with reference to both the pre- and post- at the same time, and hence from the standpoint of the unique and current frame of reference, internal standards, and values they have at such a single point in time, rather than at different times (i.e., conventional pre-test/post-test approach). Thus, the primary premise supporting this method is that, in a relatively easy fashion, it could quantify the confounding response shift bias that may occur from measuring participants experiencing different frames of reference, internal standards, and values from pre- to post-intervention. Following this approach, the presence and extent of response shift could be estimated by calculating the difference between the pre-test and then-test scores (response shift score calculation); the conventional intervention effect as the difference between the post-test and the pre-test scores (i.e., conventional score calculation); while the adjusted effect, which in plain words could be conceived as the global actual effect derived from adding response shift to the conventional intervention effect, would be operationalized as the difference between the post-test and the then-test scores (i.e., adjusted score calculation). Figure 1 illustrates the operationalization of these three types of effects (i.e., response shift, conventional, and adjusted) in the context of participants assessing their mindfulness levels at pre-test, post-test, and immediately after the post-test through the then-test.

**Fig. 1:**
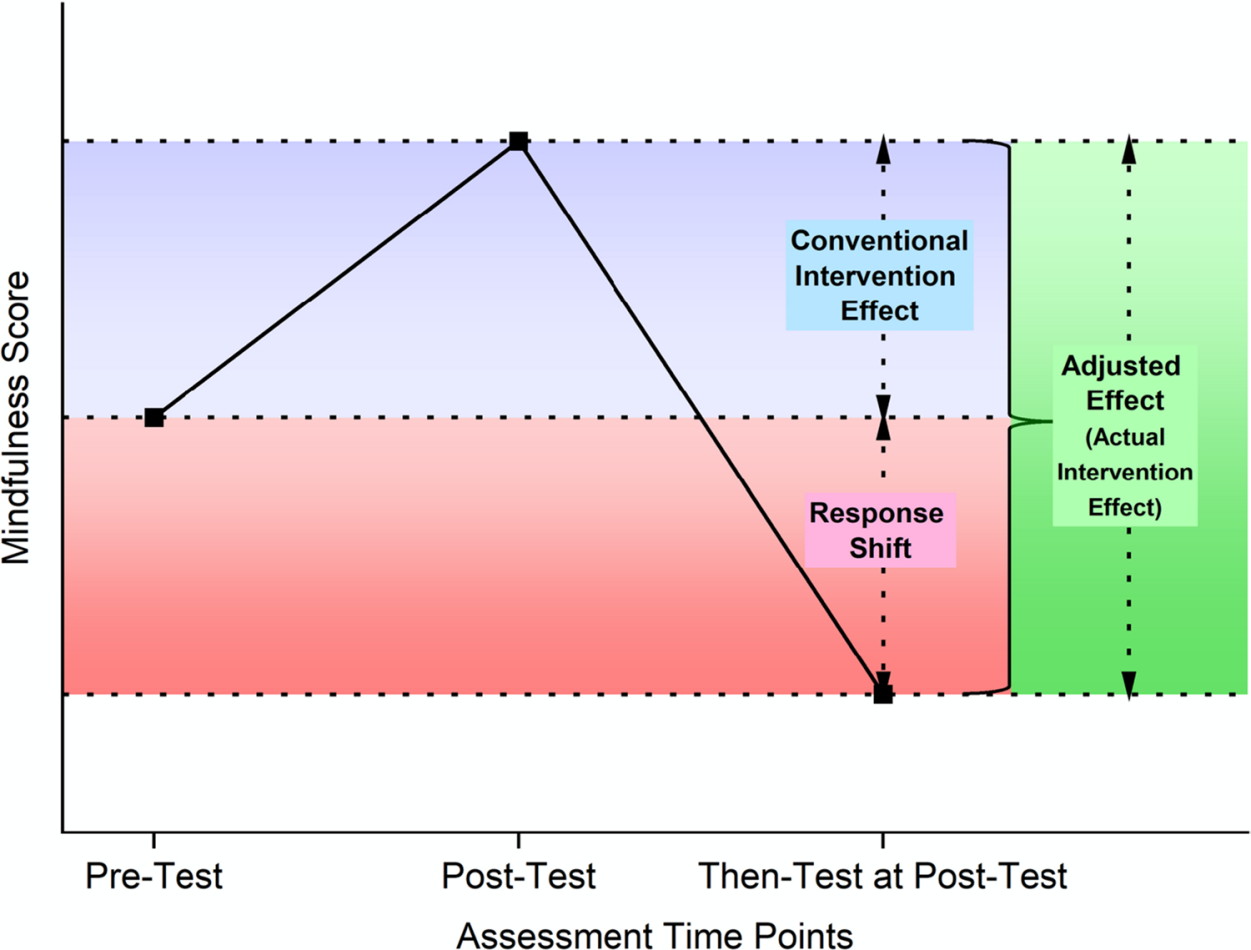
The retrospective technique *then-test* implemented in a mindfulness inventory would ask respondents, immediately after completing the post-test, to re-assess the same mindfulness items retrospectively at the time of the pre-test (before the mindfulness intervention) and, therefore, with the same frame of reference, internal standards, and values. Response shift would be operationalized as pre-test minus then-test scores, the conventional intervention effect as post-test minus pre-test scores, and the adjusted or actual intervention effect as the post-test minus then-test scores

Preliminary evidence in support of the validity of the then-test springs from a series of educational intervention studies triangulating self-reported results derived from the aforementioned score calculations (i.e., conventional, response shift, and adjusted score calculation) with those obtained from qualitative interviews, objective assessment, observational rating, and recall bias testing (Howard & Dailey, 1979; Howard et al., 1979). The results of these triangulations appear to support the contention that participants’ internal standards before and after an intervention may change, and hence, conventional effects may be confounded by response shift (Howard & Dailey, 1979; Howard et al., 1979). Overall, Howard and colleagues’ findings seemed to underpin their original hypothesis that, when using self-report instruments of change, results gathered through the retrospective then-test design (i.e., adjusted score calculation) may have greater internal validity than those yielded through the traditional approach (i.e., conventional score calculation). From the field of quality-of-life research, additional supporting evidence for the validity of the then-test has also been reported (Schwartz & Rapkin, 2012; Visser et al., 2005) and a set of guidelines for its adequate implementation has been recommended (Schwartz & Sprangers, 2010).

Despite its feasibility and easiness of application, the then-test is not exempt from relevant shortcomings (e.g., recall bias and implicit theories of change, Finkelstein et al., 2014). Notwithstanding its limitations, this method appears to have been the most widespread and accessible technique to examine response shift effects in studies with low and medium sample sizes (Finkelstein et al., 2014; Schwartz & Sprangers, 2010). In the extant literature on quality of life, significant response shift effects through the then-test have been documented in a broad range of studies investigating the potential occurrence of this phenomenon in patients suffering from serious health-related conditions such as cancer (Andrykowski et al., 2009; Dabakuyo et al., 2012; Ilie et al., 2019; Korfage et al., 2007), stroke (Ahmed et al., 2004), and multiple sclerosis (Schwartz et al., 2004). However, it appears that the existing literature does not show any studies using the then-test to determine whether response shift may have occurred and therefore confounded mindfulness measurement following mindfulness-based interventions.

A recent field study about the effects of hiking the Camino Francés on self-compassion (Steininger et al., 2023) used the then-test approach for parts of their sample as a supplementary analysis, albeit without the corresponding actual pre-test rating. Research on concepts such as self-compassion and mindfulness has thus far not been able to demonstrate the extent to which scores derived from the adjusted score calculation (i.e., adjusted scores) may differ from those obtained from the conventional score calculation (i.e., conventional scores). The current study addressed this research aim in the context of student musicians receiving curricular instruction in a mindfulness- and yoga-based program — *The CRAFT program* — at a royal conservatory of music (Posadas, 2019; Posadas & Bartos, 2022).

The newly developed CRAFT program was conceived as an art of being and holistic neuroeducational method for self-actualization, fulfillment, and well-being to address the highly cognitive, emotional, and physical demands affecting higher-education students involved in specialized educational fields such as music, fine arts, languages, and sports (Posadas, 2019). The program is grounded in the theories, practices, philosophical underpinnings, and state-of-the-art neuroscientific findings of mindfulness, yoga, emotional intelligence, and positive psychology. The acronym *CRAFT* stands for the following Spanish terms representing the five CRAFT elements: *Consciencia* (i.e., consciousness), *Relajación* (i.e., relaxation), *Atención* (i.e., attention), *Felicidad* (i.e., happiness), and *Transcendencia* (i.e., transcendence). Since 2017, the CRAFT program has been imparted at the Royal Conservatory of Music of Granada as a CRAFT-based elective subject of Mindfulness, and from 2017 onwards as both CRAFT-based elective subjects of Mindfulness and Emotional Intelligence (Bartos et al., 2021, 2022a, b; Posadas & Bartos, 2022).

The purpose of the present study was to explore the utility of the then-test to detect the presence of a potential mindfulness-based response shift occurrence during a mindfulness- and yoga-based intervention and to ascertain to what extent effect sizes may differ when adjusting for any response shift that may have occurred. To that end, we examined whether student musicians following the CRAFT program reported response shift associated with their mindfulness skills — as measured by the FFMQ — when evaluated retrospectively on such skills through a specifically developed then-test FFMQ. Additionally, we examined the extent to which participants’ FFMQ effect sizes derived from a conventional score calculation could change after being adjusted for response shift.

## Method

### Participants

Participants were higher-education student musicians, aged 18 years or older, officially enrolled in CRAFT-based elective subjects at the Royal Conservatory of Music Victoria Eugenia during the academic year 2019/2020. Participants were excluded from the study if they had received any psychological, psychiatric, and/or psychopharmacological help and if they had had previous experience with other yoga and/or meditation practices other than those learned in the CRAFT program. Following these exclusion criteria, 13 participants were excluded from the study. Thus, the final sample for the current study consisted of 31 participants, age range 19–39 years, of whom 17 were females. As can be seen in Table 1, which displays participants’ demographic characteristics, a preponderance of participants were third-year students, enrolled in the CRAFT-based elective subject of Mindfulness, and had completed their high school diploma.

**Table 1:** Participants’ demographic characteristics

Variables	
*n*	31
Age	22.71 ± 3.94
Years of musical practice	13.98 ± 3.59
Gender	
Females	17 (55%)
Males	14 (45%)
Elective subject enrolment^a^	
CRAFT-based mindfulness	23 (74%)
CRAFT-based emotional intelligence	15 (48%)
Both CRAFT-based subjects	7 (23%)
Ergonomics	3 (10%)
Music and movement	4 (13%)
English	2 (6%)
German	1 (3%)
History of Spanish music	1 (3%)
History of opera	0 (0%)
Level of education	
High school	24 (77%)
Bachelor’s degree	6 (19%)
Master’s degree	1 (3%)
Grade year	
First	4 (13%)
Second	7 (23%)
Third	15 (48%)
Fourth	5 (16%)

Values are mean $\pm$ SD or *n* (%)

^a^Participants’ enrolment in elective subjects was not mutually exclusive

### Procedure

The feasibility and preliminary effectiveness of the CRAFT program (conducted during the academic year 2018/2019) have previously been examined (Bartos et al., 2022a), and the present study reports on a subsequent implementation of the CRAFT program (conducted during the academic year 2019/2020). This longitudinal investigation was affected by the emergence of the COVID-19 pandemic, which led to a serendipitous quasi-experimental study on the applicability and perceived benefits of the program during the COVID-19 lockdown (Bartos et al., 2021). The context of this longitudinal investigation has been outlined in detail previously (Bartos et al., 2021), and here, we only describe those aspects that are relevant to the present study.

Instruction in the CRAFT program began on November 6, 2019, and was completed at the end of the teaching phase that ended on May 13, 2020. As in all other elective subjects, CRAFT-based elective classes run for 60 min, once a week, over the entire academic year. Recruitment took place at the music conservatory from January 13 to 29, 2020, via short presentations of the study objectives and requirements. Therein, 82 participants enrolled in CRAFT-based elective subjects completed Time 1 assessments which included the Time 1 FFMQ and other outcome measures as part of the longitudinal study. Due to logistical issues and delays in receiving the necessary approvals for this study, Time 1 did not constitute a true pre-test baseline assessment period but instead occurred 9 weeks into the 23-week intervention.

On March 14, 2020, after 16 weeks of program completion, both the program and the study were adapted to be continued in an online format for the remaining teaching phase due to the COVID-19 pandemic and ensuing lockdown. On June 1, 2020, 1 week after students’ final exams, participants were contacted by email and were invited to complete Time 2 evaluations online, comprising the Time 2 FFMQ, the then-test FFMQ, and other outcome measures planned for the longitudinal study. The then-test FFMQ, which was filled in by participants immediately after completing the Time 2 FFMQ, asked participants to assess their FFMQ mindfulness levels retrospectively for the Time 1 point evaluation (see “Measures” section). From the initial sample of 82 participants, 44 participants completed both the Time 2 FFMQ and the then-test FFMQ, of whom 13 participants were excluded from the analysis because they acknowledged previous yoga and/or meditation experience. For the purposes of the present response shift investigation, no appropriate control group was available. This is because Time 1 was approximately halfway through the intervention, and there was no way to ascertain that the non-CRAFT participants entered the academic year with similar levels of FFMQ scores as the CRAFT program group.

#### The CRAFT Program

The CRAFT program was implemented as part of the student’s curriculum as CRAFT-based elective subjects of Mindfulness and Emotional Intelligence during the academic year 2019/2020 at the Royal Conservatory of Music Victoria Eugenia. The dosage of delivery comprised 23 sessions of program instruction administered once a week for 60 min over the entire academic year. The first 16 sessions were imparted in a multipurpose spacious classroom at the royal conservatory, while the last seven sessions were conducted in an online format through the platform *Zoom.* The program developer (author MPP who was blinded to the study hypotheses, measures, data collection, and recruitment), a singing and pedagogy professor at the royal conservatory holding certification as an advanced mindfulness and yoga teacher (Posadas, 2019), taught all the program classes. Completion of 2 h of weekly home-based practice was encouraged among all participants in the experimental group. Instruction in both CRAFT-based elective subjects was guided by the four foundations and five elements of the program, with a preponderance of yoga and mindfulness content in the Mindfulness subject, while components of emotional intelligence, compassion, and positive psychology theory and practice were more prevalent in the Emotional Intelligence subject. Further descriptions regarding the theoretical framework of the CRAFT program can be found elsewhere (Posadas, 2019; Posadas & Bartos, 2022). Additional information with reference to the current study program procedures including an outline of the objectives, content, and practices implemented in both CRAFT-based subjects can be openly accessed in our previous quasi-experimental study (Bartos et al., 2021). By the end of the program instruction, all participants achieved the minimum 80% attendance rate required in both CRAFT-based elective subjects.

### Measures

#### Five Facet Mindfulness Questionnaire (FFMQ)

Mindfulness disposition was measured through the FFMQ (Baer et al., 2006), a 39-item survey factored into the following five subscales, each denoting a particular mindfulness-related skill: Observe (8 items; e.g., “I pay attention to sensations, such as the wind in my hair or sun on my face”), Describe (8 items; e.g., “I have trouble thinking of the right words to express how I feel about things”), Act Aware (8 reversed items; e.g., “I don’t pay attention to what I’m doing because I’m daydreaming, worrying, or otherwise distracted”), Non-judge (8 reversed items; e.g., “I think some of my emotions are bad or inappropriate and I shouldn’t feel them”), Non-react (7 items; e.g., “When I have distressing thoughts or images I am able just to notice them without reacting”). Participants’ level of agreement with each item is rated through a 5-point Likert scale oscillating between 1 (*never or very rarely true*) and 5 (*very often or always true*). Higher scores in either the total scale or a specific subscale are indicative of a higher tendency for being mindful generically or regarding a specific mindful skill respectively. The Spanish version of the FFMQ validated by Cebolla et al. (2012) with a Spanish sample was utilized. High internal consistency was reported across subscales in the present study sample ($\alpha$ ranging between 0.76 and 0.92 at pre-test).

#### The Retrospective Five Facet Mindfulness Questionnaire (Then-Test-FFMQ)

The FFMQ was adapted into a then-test-FFMQ following prescribed guidelines for then-test construction for determining response shift (Schwartz & Sprangers, 2010). In this way, the then-test FFMQ included preliminary written instructions to prompt respondents to think back to the moment just before they completed the FFMQ at Time 1, take a few seconds to recall it, and retrospectively rate their level of agreement with the then-test FFMQ items using the same FFMQ 5-point Likert scale. The then-test FFMQ items were the same as those within the FFMQ but reworded so that participants could easily refer back to the Time 1 timeframe of reference. For instance, Item 8 within the Act Aware subscale was modified as follows: “approximately four months ago, in January, I wouldn’t pay attention to what I was doing because I was daydreaming, worrying, or otherwise distracted.”

### Data Analyses

Descriptive statistics were conducted to summarize participants’ demographic characteristics and the Time 1, Time 2, and then-test FFMQ scores. Means with respective standard deviations and frequencies and percentages were computed for the continuous and categorical variables respectively.

Normality was checked through the Shapiro–Wilk test for the Time 1, Time 2, and then-test FFMQ scores. Within-group statistical significance across the conventional (i.e., Time 2-FFMQ minus Time 1-FFMQ), response shift (i.e., Time 1-FFMQ minus then-test-FFMQ), and adjusted (i.e., Time 2-FFMQ minus then-test-FFMQ) score calculations was tested through two-tailed paired sample *t*-tests if both paired dependent variables were normally distributed and by two-tailed Wilcoxon’s signed-rank tests if normality was not met by either of them at Time 1, Time 2, and then-test.

Furthermore, practical significance for each score calculation was determined through paired sample design effect size (ES) computation with respective 95% confidence interval (CI). The so-called unbiased Cohen’s *d*, or Hedges’ *g*, was calculated using the average standard deviation from each paired sample being compared, $\frac{{M}_{2}-{M}_{1}}{{SD}_{T}+{SD}_{T}/2}$ (Lakens, 2013), multiplied by a correction factor formula for reducing bias, $1-\frac{3}{8(n-1)-1}$ (Hedges & Olkin, 1985). The upper and lower ends of the 95% CIs were computed following the central *t*-distribution method (Cumming et al., 2007; Goulet-Pelletier & Cousineau, 2018; Nakagawa & Cuthill, 2007); CI = *g* $\pm$ 1.96 $\times SE$, whereby the *SE* of the CI was calculated using Becker’s (1988) formula for paired samples, *SE* = $\sqrt{\frac{2(1-{r}_{\mathrm{1,2}})}{n}+\frac{{g}^{2}}{2(n-1)}}$, being *r* the correlation coefficient between a given paired sample. Practical significance was achieved when the 95% CIs did not include the null value (Coe, 2002) and the magnitude of the effect was interpreted following Cohen (1988) ES benchmarks of small (0.20 $\le$ *d* $<0.50$), medium (0.5 $0\le$ *d* $<0.80$), and large (*d* $\ge$ 0.80).

**Fig. 2:**
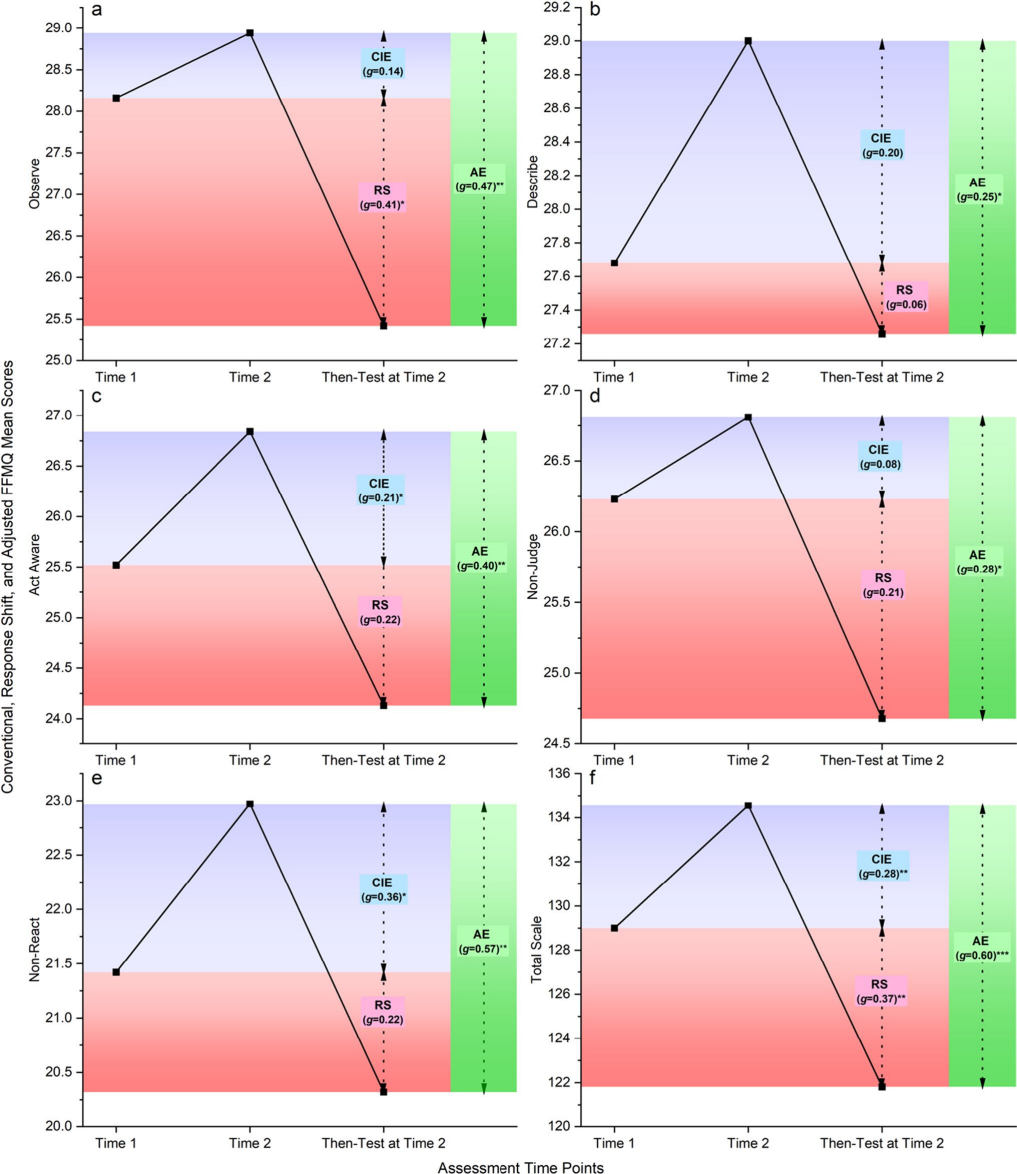
Summary of the response shift, conventional, and adjusted results including effect sizes and statistical significance within each FFMQ subscale and total scale. CIE: conventional intervention effect operationalized as Time 2-FFMQ minus Time 1-FFMQ scores; RS: response shift operationalized as Time 1-FFMQ minus then-test-FFMQ scores; AE: adjusted effect (actual intervention effect) operationalized as Time 2-FFMQ minus then-test FFMQ scores. *g*: Hedges’ *g* or Cohen’s *d* corrected for bias. *p < 0.05; **p < 0.01; ***p < 0.01

Descriptive statistics and normality analyses were computed with IBM SPSS v. 25.0 (Armonk, NY: IBM Corp. 2017) while Excel 2020 software was employed to conduct paired sample *t*-tests and calculate the ESs with respective 95% CIs. Figures (Figs. 1 and 2) were created using Origin (Pro), Version 2022 (OriginLab Corporation, Northampton, MA, USA).

## Results

Table 2 shows the FFMQ results including descriptive statistics and both statistical and practical significance across the conventional, response shift, and adjusted score calculations. Data were normally distributed for all FFMQ subscales and total scale at Time 1, Time 2, and then-test except for the Observe subscale at Time 1.

**Table 2:** Descriptive statistics and practical and statistical significance across three FFMQ score calculations

Conventional score calculation (Time 2-FFMQ minus Time 1-FFMQ)										
FFMQ	Time 1		Time 2		*t/z*^*d*^	*p*	*g*	*SE (g)*	95% CI (g)	
	*M*	*SD*	*M*	*SD*					LL	UL
Observe	28.16^d^	4.55^d^	28.94	6.26	1.16^d^	0.24^d^	0.14	0.10	-0.06	0.34
Describe	27.68	6.42	29.00	6.90	1.74	0.09	0.20	0.12	-0.03	0.42
Act Aware	25.52	5.61	26.84	6.74	2.11	**0.04**	**0.21**	0.10	0.01	0.41
Non-judge	26.23	6.62	26.81	7.26	0.48	0.63	0.08	0.17	-0.26	0.42
Non-react	21.42	4.65	22.97	3.95	2.26	**0.03**	**0.36**	0.16	0.04	0.68
Total	129.00	17.74	134.55	20.78	3.10	**<0.01**	**0.28**	0.10	0.10	0.47
Response shift score calculation (Time 1-FFMQ minus then-test-FFMQ)										
FFMQ	Time 1		Then-Test		*t/z*^*d*^	*p*	*g*	*SE(g)*	95% CI (g)	
	*M*	*SD*	*M*	*SD*					LL	UP
Observe	28.16^d^	4.55^d^	25.42	8.51	2.50^d^	**0.01**^d^	**0.41**	0.13	0.16	0.67
Describe	27.68	6.42	27.26	6.61	0.60	0.55	0.06	0.11	-0.15	0.28
Act Aware	25.52	5.61	24.13	6.61	1.66	0.11	0.22	0.14	-0.04	0.49
Non-judge	26.23	6.62	24.68	7.92	1.23	0.23	0.21	0.17	-0.13	0.55
Non-react	21.42	4.65	20.32	5.29	1.47	0.15	0.22	0.15	-0.08	0.52
Total	129.00	17.74	121.81	21.13	3.06	**<0.01**	**0.37**	0.13	0.12	0.61
Adjusted score calculation (Time 2-FFMQ minus then-test-FFMQ)										
FFMQ	Time 2		Then-Test		*t*	*p*	*g*	*SE(g)*	95% CI (g)	
	*M*	*SD*	*M*	*SD*					LL	UP
Observe	28.94	6.26	25.42	8.51	3.51	** < 0.01**	**0.47**	0.14	0.20	0.74
Describe	29.00	6.90	27.26	6.61	2.70	** < 0.05**	**0.25**	0.10	0.06	0.45
Act Aware	26.84	6.74	24.13	6.61	3.25	** < 0.01**	**0.40**	0.14	0.14	0.67
Non-judge	26.81	7.26	24.68	7.92	2.50	** < 0.05**	**0.28**	0.12	0.05	0.51
Non-react	22.97	3.95	20.32	5.29	3.44	** < 0.01**	**0.57**	0.18	0.22	0.91
Total	28.94	6.26	121.81	21.13	4.92	** < 0.01**	**0.60**	0.15	0.31	0.89

Bold font denotes significance

*FFMQ*: Five Facet Mindfulness Questionnaire; *g*: Hedges’* g* = Cohen’s *d* corrected for bias; *SE (g)*: standard error of *g*; *CI*: confidence interval of *g*; *LL*: lower limit; *UL*: upper limit

^d^*M*, *SD*, *z* score, and* p*-value involving a non-normally distributed variable*.* Statistical significance is achieved if *p* $<0.05$ and was derived from a two-tailed paired* t*-test if normality was met or a Wilcoxon’s signed-rank test when a non-normally distributed variable was entered in the score computation; practical significance is determined through effect sizes (Hedges’ *g*), achieved if CIs do not include the null value of zero, and interpreted following Cohen’s (1988) *d* benchmarks: small (0.20 $\le$ *d* $<0.50$), medium (0.5 $0\le$ *d* $<0.80$), and large (*d* $\ge$ 0.80)

To test whether student musicians following the CRAFT program would report a mindfulness-based response shift occurrence, we examined whether the within-group difference between the Time 1-FFMQ and then-test-FFMQ mean scores (i.e., response shift score calculation) for each subscale and total scale was statistically and practically significant. For the response shift score calculation, statistically and practically significant response shift trending toward a medium effect was found for the Observe FFMQ subscale, *z* = 2.50, *p* = 0.013, *g* = 0.41, 95% CI (0.16, 0.67) and total FFMQ scale, *t*(31) = 3.07, *p* $<0$.01, *g* = 0.37, 95% CI (0.12, 0.61). However, response shift was neither statistically nor practically significant for the FFMQ subscales of Describe, Act Aware, Non-judge, and Non-react.

In the conventional score calculation, there were statistically and practically significant increases for the Act Aware, *t*(31) = 2.11, *p* = 0.04, *g* = 0.21, 95% CI (0.01, 0.41) and Non-react, *t*(31) = 2.26, *p* = 0.03, *g* = 0.36, 95% CI (0.04, 0.68) FFMQ subscales as well as the total FFMQ scale, *t*(31) = 3.11, *p* $<0$.01, *g* = 0.28, 95% CI (0.10, 0.47) with a small effect size. No significant conventional scores were found for the Observe, Describe, and Non-judge FFMQ subscales.

To determine the extent to which participants’ conventional scores could change after being adjusted for a potential mindfulness-based response shift occurrence, practical and statistical significance was examined for the within-group difference between the Time 2-FFMQ and then-test-FFMQ mean scores (i.e., adjusted score calculation) for each subscale and total scale. In the adjusted score calculation, statistical and practical significance was reported for all subscales and total scale of the FFMQ with a medium effect size for the Non-react, *t*(31) = 3.44, *p* $<0$.01, *g* = 0.57, 95% CI (0.22, 0.91) FFMQ subscale and total FFMQ scale, *t*(31) = 4.92, *p* $<0$.001, *g* = 0.60, 95% CI (0.31, 0.89); a close to a medium effect for the Observe, *t*(31) = 3.51, *p* $<0$.01, *g* = 0.47, 95% CI (0.20, 0.74) and Act Aware, *t*(31) = 3.26, *p* $<0$.01, *g* = 0.40, 95% CI (0.14, 0.67) FFMQ subscales, and a small effect size for the Describe, *t*(31) = 2.70, *p* = 0.01, *g* = 0.25, 95% CI (0.06, 0.45) and Non-judge, *t*(31) = 2.50, *p* = 0.02, *g* = 0.28, 95% CI (0.05, 0.51) FFMQ subscales. Figure 2 summarizes the current study results through the same schema that was utilized in Fig. 1 to illustrate the operationalization of the response shift, conventional, and adjusted effects. After adjusting for a potential mindfulness-based response shift occurrence, the reported effect sizes increased for all FFMQ subscales and the total scale. In comparison to the effect sizes derived from the conventional score calculation, those computed through the adjusted score calculation appear to be nearly two times greater for the total FFMQ scale and the FFMQ Non-react and Act Aware subscales, and over three times higher for the FFMQ Observe and Non-judge subscales.

## Discussion

The current study explored the utility of the then-test to determine whether there was a mindfulness-based response shift occurrence in participants of a mindfulness- and yoga-based intervention (e.g., the CRAFT program), and to what extent effect sizes in mindfulness might change when making the pertinent response shift adjustments. The results provided evidence of a significant response shift effect for the FFMQ Observe subscale and total scale score but not for the Describe, Act Aware, Non-judge, and Non-react subscales. The adjusted scores in all FFMQ subscales and total FFMQ scale achieved significance and larger effect sizes than the conventional results, for which only the FFMQ Act Aware and Non-react subscales and the total scale were significant.

In alignment with the conventional and adjusted results of the current study, significant improvements in dispositional mindfulness as measured by the FFMQ were also found in previous studies examining the effectiveness of the CRAFT program for enhancing the well-being of higher-education translation students (Cásedas et al., 2022) and student musicians (Bartos et al., 2022a) as well as other mindfulness-based interventions conducted with student musicians (Czajkowski et al., 2020; Steyn et al., 2016). However, these findings were mixed in terms of the FFMQ subscales that achieved significance, which may be explained by the differential weekly dosage of program delivery administered in each study. For instance, with 1 h of program delivery per week, significant within-group improvements were reported for the subscales of Act Aware and Non-react in the conventional results of the current study, Observe and Describe in the feasibility study of Bartos et al. (2022a), and Observe and Non-judge in the study of Steyn et al. (2016). By contrast, in the studies of Cásedas et al. (2022) and Czajkowski et al. (2020), where the weekly dose of program delivery was 2 and 2–2.5 h respectively, significant improvements were found for all FFMQ subscales. The findings from these latter studies align with the adjusted results reported in the current study as all FFMQ subscales turned out to be significant and showed greater effect sizes compared to those conventionally calculated. For instance, after adjusting for the potential response shift that may have occurred, the effect sizes for the FFMQ subscales of Observe and Non-react, as well as the total FFMQ scale, increased from small to medium. These adjusted scores, in turn, raise questions as to whether the actual effect in mindfulness, or even in other self-reported outcome measures that may have been engendered by the aforementioned mindfulness-based programs and other similar interventions using equivalent measures, may have been in actuality greater than what was reported. In order to answer these questions, it is necessary to find out whether and to what extent mindfulness-based interventions in the first place may induce response shift.

As indicated above, the findings of the current study are partially supportive of a potential mindfulness-based response shift occurrence specifically detected through the then-test for the total FFMQ scale and the FFMQ Observe subscale. In addition, despite the FFMQ mean scores in the response shift score calculation for the other subscales not being significant, they exhibited a downward Time 1–then-test pattern (i.e., lower then-test FFMQ scores than Time 1-FFMQ scores) leaning toward the response shift direction. Explanatory reasons for the potential presence of response shift bias in the current study may require further inquiry on the axial and most prominent components characterizing both response shift and yoga and mindfulness theory and practice as well as their interconnections. Drawing from their definition of response shift, Sprangers and Schwartz (1999) proposed a theoretical response shift model whereby perceived quality of life could be influenced by the occurrence of response shift’s recalibration, reprioritization, and reconceptualization from individual health-related changes (i.e., *catalysts*). In this model, one’s response shift experience, and hence ultimately one’s perception of quality of life, could be also directly or indirectly influenced by a series of individual traits or *antecedents* (e.g., sociodemographic characteristics, spiritual lineage) and the types of *mechanisms* (e.g., coping strategies that could also in turn act as *catalysts*) adopted to adapt to the *catalyst*. The most relevant adjustment to this original model was the inclusion of changes in cognitive appraisal (Rapkin et al., 2017; Rapkin & Schwartz, 2004, 2019) as a key underlying factor influencing both response shift and perceived quality of life. The authors contended that, in any self-reported quality of life process, response shift could be conceptualized as a change in one’s cognitive appraisal depending upon reconceptualization, recalibration, and reprioritization. If we were first to extrapolate the initial response shift model of Sprangers and Schwartz (1999) to the context of a mindfulness- and yoga-based intervention, replacing quality of life for dispositional mindfulness, it could be argued that this intervention could function as both the *catalyst* and the *mechanism* that may influence response shift from changes in internal standards, values, and meanings, ultimately affecting, in turn, perceived mindfulness. In this milieu, a relevant underlying reflection may be whether response shift would not necessarily be only a nuisance for measurement but actually an intended aspect of the intervention, or essentially, the transformative phenomenological lived experience that may occur from following such an intervention. In one of the first publications examining the response shift bias, Collins et al. (1985) referred to the subject in the following terms:

The response shift bias affects pretest-posttest designs in which a treatment group receives an intervention intended to alter behavior. When a behavioral intervention alters not only the treatment subjects’ behavior, but also their awareness of the behavior and their judgment about their own level of skill, the treatment subjects may undergo a change in their interpretation of the anchors of a response (p. 301).

It could thus be argued that the first two plausible response shift-based contingencies, implied by Collins et al. (1985) as a change in both participants’ behavior and their awareness of it due to the intervention, are in close alignment with the typical effects experienced by mindfulness and yoga practitioners. For instance, the method of yoga through its different paths (e.g., *Raja*, *Bhakti*, *Karma*, *Jnana*; Lidell et al., 1983) prescribes a wide range of ethical, postural, breathing, meditative, devotional, self-less, and self-inquiry practices for self-realization that might bring about a change in an individual’s awareness, behavior, perspective, meaning, and values (Sullivan et al., 2018), a shift that also transversally mirrors, by definition, the main response shift components identified by Sprangers and Schwartz (1999). By following this method, yoga practitioners might progressively learn to dis-identify with the ever-changing reality from which the dual experience of suffering and pleasure emerges (Lidell et al., 1983; Satyananda, 2013; Sullivan et al., 2018). This entails the cultivation of higher values, virtues, capabilities, and states such as heightened awareness, equanimity, unconditional love, truthfulness, non-violence, selfless service detached from the outcome, eudaimonic happiness, and living a meaningful and purposeful life for the ultimate purpose of realizing an ever-lasting reality or pure consciousness (Lidell et al., 1983; Satyananda, 2013; Sullivan et al., 2018). In the CRAFT program, the development of these attributes for progressively becoming less identified with the changing reality along the path of self-actualization is a crucial aspect that is nurtured through both yoga and mindfulness practices within the learning experience of the five CRAFT elements (i.e., consciousness, relaxation, attention, happiness, and transcendence) and, specifically, through meditation techniques (Bartos et al., 2021, a, b; Posadas & Bartos, 2022). In the modern mindfulness literature, such a dis-identification process, particularly with the changing phenomena of thoughts and emotions, has been explained through the lenses of a mindful metacognitive mechanism of reperceiving or decentering (Shapiro et al., 2006) triggered by mindful meditative practices.

Through reperceiving, yoga and mindfulness practitioners may learn to dis-identify with the content of their consciousness (e.g., raising thoughts and emotions) by observing them non-judgmentally (Chiesa et al., 2013; Gard et al., 2014; Garland et al., 2009; Shapiro et al., 2006; Sullivan et al., 2018). Concomitantly, under such a state of meta-awareness, new creative insights and perspectives might emerge and eventually serve for re-construing one’s primary appraisals with a more adaptive approach (Garland et al., 2009, 2011). Therefore, the reperceiving process could represent in itself not only a change in one’s cognitive appraisal, paradoxically, by virtue of a non-judgmental observation or witnessing, but also a substantial shift in terms of the frame of reference or perspective from which the subject perceives or identifies themselves, and ultimately the actual conception of themselves. As Shapiro et al. (2006) indicates with reference to reperceiving, “[t]hrough this change in perspective, identity begins to shift from the contents of awareness to awareness itself” (p. 379). In this process, the non-judgmental attitude of witnessing is key so that full attention without conditioning is given to the object of concentration. As Krishnamurti (2019) posits, such full attention ultimately gives rise to the unity between the observer and the observed, or in other words, the observer becomes the observed, such realization being the beginning of freedom and the end of duality, conflict, and division. Thus, in reconciling these reasonings with the above-mentioned response shift theoretical frameworks (Rapkin et al., 2017; Rapkin & Schwartz, 2004, 2019; Sprangers & Schwartz, 1999), it could be postulated that, if mindfulness- and yoga-based meditative practices stimulate reperceiving and this, in turn, may result in changes in one’s cognitive appraisal and frame of reference, then such mindfulness- and yoga-based experiences may in itself constitute a response shift phenomenon.

Another possible explanation for the occurrence of response shift in the current study may be found in the context of the Dunning-Kruger effect and its emergent paradox (Kruger & Dunning, 1999). According to this well-known effect, new learners of a particular skill would tend to overestimate their levels of competence during the early phase of skill acquisition, followed by a re-adjustment toward accurate skill estimation or even to the point where there may be a period of underrating their level of expertise as it continues to increase (Bradley et al., 2022; Dunning, 2011; Kruger & Dunning, 1999). Through the original set of experiments that shed light on the potential existence of this effect, Kruger and Dunning (1999) systematically found a *miscalibration* between the actual and perceived performance results reported by low- and high-scoring participants undergoing tests of humor, reasoning, and grammar. Such a miscalibration was revealed by the fact that participants scoring in the lowest quartile believed their level of competence was higher than 62% of their peers, despite actually only being higher than 12% of them. By contrast, participants scoring in the highest quartile tended to underestimate their abilities relative to the other participants, yet the magnitude of their miscalibration was reported to be nearly four times lower than those scoring in the lowest quartile.

In explaining the high miscalibration of the least competent participants, the authors argued that unskilled individuals may lack the necessary metacognitive abilities to self-realize their own incompetence and consequently self-appraise their level of skill accurately. In support of their reasoning, Dunning (2011) highlighted that the metacognitive process of judging one’s level of ability in a particular task is dependent on the very same skills required to accomplish the actual task. Thus, on the basis of their findings and subsequent explanatory premises, it might be possible that the novice mindfulness and/or yoga participants of the current study may have overestimated their mindfulness skills at Time 1, while moving toward more accurate estimation or potentially underestimating their skill level at Time 2 (end of the intervention). Therefore, the miscalibration found by Kruger and Dunning (1999) may explain and align to a certain extent with the possible recalibration-based response shift occurrence that participants in the current study may have experienced in terms of their mindfulness skills.

Moreover, as Kruger and Dunning (1999) and Dunning (2011) underlined, individuals’ enhancements in a particular skill through training, along with its accompanied metacognitive development, would help them realize their initial limited competency they might have been unaware of. On the one hand, this argument appears to support the relevant role that mindfulness and/or yoga-based interventions — which have been conceptualized as sound instigators of a metacognitively enhanced experience (Garland et al., 2009, 2011; Shapiro et al., 2006) — may play in promoting such realization. On the other hand, it would also further bolster the consideration of the Dunning-Kruger effect as a potential mechanism influencing, underlying, and/or explaining response shift, particularly as far as its recalibration component is concerned. This may be because the training process involved in learning a particular ability, in turn, could somewhat signify in itself a catalyst for changing learners’ internal standards relative to the specific construct domains of that particular ability they are trained for. In other words, could it in actuality constitute a recalibration response shift experience that would calibrate what was in the early stages miscalibrated within and beyond the boundaries of self-reported response biases versus actual objective reports?

Although the results of the present study appear to bolster the potential occurrence of response shift from mindfulness and yoga exposure (e.g., the CRAFT program) amongst student musicians, we did not statistically uncover such a possibility for the Describe, Act Aware, Non-judge, and Non-react subscales. The absence of significant response shift in these FFMQ subscales could be due to various reasons. First, the Time 1 assessment for the FFMQ was not an actual pre-test, for it occurred halfway through the intervention after participants had already received 3 months of program instruction. For this reason, the Time 1-FFMQ  may have been a more challenging period to recall for participants than the period immediately before embarking upon the intervention. This may be because the intervention itself may remain within participants’ memory as the actual catalyzer highlighting the beginning of a contrast, within which and as a result of which, participants’ frame of reference, meanings, and values in relation to their mindfulness skills may have changed. Concomitantly, this absence of an actual pre-test may have limited the ability to find significant results for all FFMQ subscales in the conventional score calculation of the current study because it is unknown what participants’ FFMQ conventional scores would have been at pre-test, 3 months before Time 1 assessments. Moreover, as indicated above, the CRAFT program dosage of delivery did not exceed 1 h per week, which, in addition to possibly affecting the conventional scores, may have also hampered the occurrence and/or magnitude of the current study potential response shift effect. As underlined by Howard et al. (1979) and further supported by Collins et al., 1985; see aforementioned quotation), response shift could be intensified if the aim of the intervention is to change participants’ conception or awareness of the construct being measured, which for a mindfulness intervention may soundly revolve, though arguably not exclusively, around mindfulness. Nonetheless, 1 h of CRAFT instruction per week may not have been enough to modify participants’ awareness and understanding of mindfulness, and therefore engender and/or potentiate a mindfulness-based response shift occurrence. Furthermore, the lack of significant response shift and conventional results in some subscales could be also related to the differential emphasis given to some mindfulness and yoga practices, concepts, and attitudes relative to other ones; and misalignments of the actual program delivery and/or participants’ home-based practice with the originally planned CRAFT program content and objectives (Bartos et al., 2022a). However, even if response shift itself was not significant for some of the facets, their effect sizes after adjusting for it were higher than when conventionally computed.

### Limitations and Further Research

There are various limitations that can be identified in this study. First, soliciting participants to undertake a retrospective assessment through the then-test could be subject, in turn, to a possible recall bias (Finkelstein et al., 2014; Schwartz et al., 2004), which may lead to a scanty recollection of their pre-intervention states. According to implicit theories of change (Finkelstein et al., 2014; Norman, 2003), the inability to remember or deficiently recollect such an initial state may prompt participants to make erroneous assumptions about how this state actually was through a backwards extrapolation from their current state (e.g., “I received mindfulness instruction and I feel my ability to concentrate has improved, thus, before the training, my attention skills must have been at a lower level”). In the current study, this plausible recall bias may have been further complicated by the fact that there was not an actual pre-test but a Time 1-FFMQ assessment occurring halfway through the intervention. Second, the low dosage of CRAFT program delivery per week appears to be a critical limitation affecting the conventional, response shift, and adjusted effects estimated in the current investigation. Therefore, based on previous discussion (see above), it would be recommended that further research should incorporate higher doses of program delivery of at least 2 h of instruction per week. The third shortcoming relates to the low sample size of this study, as previous researchers have required a high sample size for examining response shift through different methods relying on psychometrics and measurement invariance testing (Krägeloh et al., 2018). However, the then-test has been distinguished as a recommended method applicable for examining response shift within the low to medium sample size range (Finkelstein et al., 2014; Howard et al., 1979; Sprangers et al., 1999) to which the current study sample belongs.

A fourth relevant limitation was the lack of availability of an effective control group uninvolved in any mindfulness- and/or yoga-related activities that had undergone an actual pre-test assessment. This would have enabled meaningful between-group comparisons related to the three types of score calculations (i.e., response shift, conventional, and adjusted) and requires further examination through experimental controlled longitudinal studies. Fifth, in the current study, out of the 31 participants enrolled in CRAFT-based elective subjects, eight participants were exclusively attending the CRAFT-based elective subject of Emotional Intelligence that emphasizes positive psychology and emotional intelligence components. Although both yoga and mindfulness were also taught as part of the curriculum of this elective subject, for these eight participants, their exposure to these practices and underlying theories may have been lower compared to the other participants. Whether such a differential emphasis in program component instruction between the two CRAFT-based elective subjects could result in diverse response shift effects among participants following the mindfulness, emotional intelligence, or both CRAFT-based subjects simultaneously may deserve additional examination in future investigations. Sixth, due to the unavailability of ordinal-to-interval conversion algorithms for the Spanish version of the FFMQ, score calculations were implemented using ordinal summary scores, which would have diminished measurement precision (Medvedev & Krägeloh, 2022). For the English-language version of the instrument, conversion tables have been published previously (Medvedev et al., 2017), which need to be generated for other versions of the instrument as well. Seventh, the inclusion of qualitative methods such as semi-structured interviews could have been an additional asset to either clarify, confirm, or call into question the quantitative results derived from the then-test. This procedure would allow researchers to capture a greater understanding of the potential response shift that participants may have experienced in relation to their mindfulness- and/or yoga-based lived experience through the program in addition to their conventional, adjusted, and response shift mindfulness scores. Moreover, the incorporation of a qualitative component could serve to explore whether recall bias or conflicting assumptions from participants not remembering their initial pre-intervention state may occur. Furthermore, the current study was disrupted by the emergence of the COVID-19 pandemic and ensuing national lockdown. Therefore, it is uncertain whether and to what extent this unprecedented event loaded with highly demanding and stressful circumstances may have incurred some form of reconceptualization, reprioritization, and recalibration in relation to the FFMQ items and therefore further confounded measurement. Lastly, it appears that within the extant literature on response shift and particularly mindfulness, there are no studies examining the potential implications of the Dunning-Kruger effect (Kruger & Dunning, 1999) in the context of subjective self-reported assessment and response bias with regard to mindfulness. As has been discussed, it seems that this effect may be contributing to some response shift effects. However, to what extent it applies to the present study is unclear without more detailed data and therefore further research is needed to ascertain this.

Despite these limitations, this study revealed the utility of the then-test to potentially detect and quantify a mindfulness-induced response shift occurrence in participants following a mindfulness- and yoga-based intervention. In the current study, response shift was significant for the total FFMQ scale and the Observe subscale but not for the remaining FFMQ subscales. After adjusting the conventional intervention results for response shift, the effect sizes for all FFMQ subscales and total scale were amplified. Although these findings show partial evidence of response shift within participants following mindfulness- and yoga-based training, whether response shift may have actually occurred or not as a result of receiving such instruction requires further examination. To that end, future mindfulness- and/or yoga-based interventions using the then-test as part of an experimental controlled design are needed. In light of the limitations of the current study, we also encourage that these interventions administer a weekly dose of program delivery of at least 2 h and include a qualitative component of retrospective inquiry to either confirm, clarify, or dispute then-test findings of dispositional mindfulness. In addition, we endorse that the then-test be extended to other health and well-being instruments, other than those measuring dispositional mindfulness, to explore in a systematic fashion whether response shift may occur in other relevant outcome variables as a result of mindfulness- and yoga-based interventions.

## Data Availability

The datasets presented in this article are not readily available because it was informed in the participants’ consent form that all data collected for this study would be used anonymously for their analysis and further publication as an aggregate data, but never individually. The participants recruited in this study constitute a small sample from just one institution (Royal Conservatory of Music Victoria Eugenia, Granada, Spain) and from specific low-class-size courses. Therefore, although participants used an alphanumeric code to safeguard their anonymity, in some instances, they could be potentially identifiable. Nevertheless, any queries about the availability of the datasets to be used for research purposes can be directed to LJB, javier.bartos@autuni.ac.nz.
